# Critical assessment of digital PCR for the detection and quantification of genetically modified organisms

**DOI:** 10.1007/s00216-018-1010-1

**Published:** 2018-03-24

**Authors:** Tigst Demeke, David Dobnik

**Affiliations:** 1 Canadian Grain Commission, Grain Research Laboratory, 1404-303 Main Street, Winnipeg, MB R3C3G8 Canada; 2 Department of Biotechnology and Systems Biology, National Institute of Biology, Večna pot 111, 1000 Ljubljana, Slovenia

**Keywords:** Digital PCR, Droplet digital PCR, Chip-based digital PCR, Genetically modified organisms, Quantification

## Abstract

The number of genetically modified organisms (GMOs) on the market is steadily increasing. Because of regulation of cultivation and trade of GMOs in several countries, there is pressure for their accurate detection and quantification. Today, DNA-based approaches are more popular for this purpose than protein-based methods, and real-time quantitative PCR (qPCR) is still the gold standard in GMO analytics. However, digital PCR (dPCR) offers several advantages over qPCR, making this new technique appealing also for GMO analysis. This critical review focuses on the use of dPCR for the purpose of GMO quantification and addresses parameters which are important for achieving accurate and reliable results, such as the quality and purity of DNA and reaction optimization. Three critical factors are explored and discussed in more depth: correct classification of partitions as positive, correctly determined partition volume, and dilution factor. This review could serve as a guide for all laboratories implementing dPCR. Most of the parameters discussed are applicable to fields other than purely GMO testing.

**Graphical abstract:**
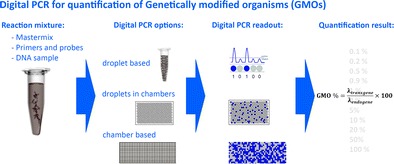
There are generally three different options for absolute quantification of genetically modified organisms (GMOs) using digital PCR: droplet- or chamber-based and droplets in chambers. All have in common the distribution of reaction mixture into several partitions, which are all subjected to PCR and scored at the end-point as positive or negative. Based on these results GMO content can be calculated.

There are generally three different options for absolute quantification of genetically modified organisms (GMOs) using digital PCR: droplet- or chamber-based and droplets in chambers. All have in common the distribution of reaction mixture into several partitions, which are all subjected to PCR and scored at the end-point as positive or negative. Based on these results GMO content can be calculated.

## Introduction

Genetically modified organisms (GMOs) have already passed a two-decade milestone of presence on the market, and the number of GMOs as well as the worldwide area planted with biotech crops continues to increase steadily [1]. Many countries regulate the cultivation and trade of GMOs [2, 3], whereas others have at least some kind of authorization system and/or labelling requirements in place for GMOs present in food and feed chains [3, 4]. Labelling thresholds are relatively low in some countries; for example, in the European Union (EU) Regulation (EC) 1829/2003 [5] has set the labelling threshold for food products that contain, consist of or are produced from authorized GMOs at 0.9%. For feed samples there is an even lower threshold of 0.1% for GMOs, for which authorization is either pending or has expired (so-called low-level presence) [6]. Additionally, there is zero tolerance for unapproved GMOs in EU countries and some other countries. As a consequence, sensitive and accurate methods must be used for GMO detection and quantification to check product labelling compliance with the legislation. During the last 20 years, several approaches have been developed for GMO detection and quantification, and these are generally divided into two groups: protein-based and DNA-based methods. Because of several performance parameters, DNA-based methods are more widely accepted and used. One of the most promising DNA-based methods is digital polymerase chain reaction (dPCR), which is critically reviewed in this article.

## Overview of different technologies used for GMO detection

### Protein-based methods

The most commonly used protein-based methods for detection and quantification of GMOs are enzyme-linked immunosorbent assay and use of lateral flow devices (LFDs; or lateral flow strips) [7]. Although protein-based methods are used less often than DNA-based methods, they have some advantages. For instance, LFDs are inexpensive, are simple to use, and provide quick detection of the presence or absence of proteins coded by the inserted GMO gene. They can be used on-site (e.g. at grain elevators) with minimum expertise and equipment requirements (e.g. in the format of a portable immunosensor as suggested for on-site GMO monitoring) [8]. LFDs are also available in a comb format for the detection of multiple GMOs. They are also useful for screening of plant seedlings for the presence of specific GMO traits, and can also be used for analysis of grain samples in which the proteins are not sheared. The quantitative aspect of protein-based methods is addressed with enzyme-linked immunosorbent assay, which provides quantitative test results using known reference standards and optical plate readers [9]. Protein-based methods have been used for verification of the identity preservation system for non-GMO grains (e.g. soybean). Nevertheless, protein-based methods also have some drawbacks. Development of antibodies is expensive and time-consuming, in some instances different levels of protein expression can occur, variation in protein content can occur in different tissues and cells, and perhaps most importantly, there is a lack of availability of protein-based methods for all GMO events [7, 10, 11]. All protein-based methods are specific only for the protein coded by the inserted GMO construct, and are not specific for the GMO trait. Proteins are also more sheared than DNA in processed samples (e.g. food products). Thus, protein-based methods are not suitable for identification and quantification in processed samples.

### DNA-based methods

Polymerase chain reaction (PCR) is the leading DNA-based method for GMO detection. A second generation of PCR—that is, real-time quantitative PCR (qPCR)—has been the gold standard for GMO trait detection and quantification for more than a decade. The PCR-based GMO detection analysis approach usually starts with qualitative screening for the presence of genetic elements commonly found in GMOs, such as cauliflower mosaic virus 35S promoter (p35S) and nopaline synthase terminator (tNOS) [12, 13]. Qualitative screening can efficiently reduce the number of subsequent analyses [13]. More and more GMO traits are appearing that do not contain any of the five most common screening elements (p35S, tNOS, ctp2-cp4-epsps, bar and pat), meaning that additional reactions must be performed for their detection/identification. Construct-specific PCR test methods are based on primers and probes targeting a particular genetic construct (junction of two transgenic elements inside the transgenic cassette), and provide more specific information compared with screening elements. Sets of screening and construct-specific PCR methods have been validated through ring trials [14]. Event-specific qPCR provides the most accurate identification of a particular GMO event. Validated event-specific qPCR GMO detection methods are available on the EU Reference Laboratory for GM Food and Feed website [15], and can be used for quantitative analysis when used simultaneously with plant endogene assay.

In terms of qualitative PCR assays, prespotted plates have been effectively used for detection of multiple GMOs in various crop plants [16, 17]. These are plastic plates used in qualitative PCR with primers and probes for selected assays predispensed in wells. Hands-on time is shortened because only one reaction mixture needs to be prepared per sample, which is then distributed over several wells [16]. Element-specific, taxon-specific and event-specific prespotted plates can be prepared and used for the detection of multiple GMOs. This approach can substitute the screening phase. However, quantification might still be necessary, if specific GMOs are detected. With the increasing number of GMO events in food and feed products, the capacity to detect multiple GMO events in one PCR will speed up testing and increase cost-effectiveness [18–20]. However, multiplexing with PCR is challenging as a result of sequence-dependent interaction of primers and preferential amplification of some targets. Ligation-based multiplex qPCR was used for the determination of eight genetically modified maize events, and was reported to offer many advantages over traditional multiplex PCR [21]. In reality, multiplex qPCR has not been routinely used by GMO testing laboratories.

There are other DNA-based detection methods, which also provide a multiplex detection system, such as capillary gel electrophoresis and different kinds of microarrays (both reviewed by Milavec et al. [22]), but these methods are not used for routine GMO analysis. Direct genomic DNA hybridization, without any amplification, to a high-density microarray was used for GMO monitoring [23]; however, the method has limited sensitivity and quantitative ability. Microarray-based methods have not been routinely used for testing of GMO events as their validation is time-consuming and additional equipment is needed for hybridization and analysis [22].

Loop-mediated isothermal amplification (LAMP) is a simple qualitative detection method which provides results in less than half an hour [24, 25]. One of the drawbacks of LAMP is the complexity of primer design, which relies on a known sequence that is either hard to acquire for patented GMO constructs or differs between the GMO construct tested and the one patented. A quantitative LAMP method for GMO detection has also been suggested [26], but its performance is not in the range of qPCR and it does not comply with the minimum performance requirements for analytical methods for GMO testing [27]. Capillary-array-based LAMP for multiplex visual detection of nucleic acids has been suggested for monitoring of GMOs [28]. The system provides the ability to detect multiple nucleic acids in a single test. Seven frequently detected transgenic elements and five endogenous reference genes were detected with high specificity and sensitivity. Although there are many publications on the use of LAMP for GMO detection, the LAMP system is not routinely used for GMO testing. Wide applicability of the LAMP system needs to be evaluated in collaborative studies, and there is already one report of such a study [29]. On the basis of this report it can be expected that LAMP will be used primarily as a screening tool, if it enters into routine GMO testing.

Another technology in the spotlight is next-generation sequencing (NGS). This technology is an alternative method for detection of authorized and unauthorized GMOs in food and feed chains [30–32]. There are different approaches for the detection of GMOs with NGS. The two common formats are whole-genome sequencing and sequencing after enrichment. Whole-genome sequencing was shown to be applicable for detection and characterization of GMOs and derived products; however, there are still problems with sensitivity for all targets [33]. To increase sensitivity, DNA enrichment approaches can be coupled with NGS [34], and such a combination can allow reliable identification of authorized and unauthorized GMOs [35]. The main drawback of NGS is currently the price and complex data analysis, which restricts its routine use.

Digital PCR (dPCR) technology emerged as a third-generation PCR technique. It was first described as a concept in the 1990s [36, 37] with a rather simple idea. When a sample at limiting dilution (meaning at low concentration) is amplified, some end-point results are positive and others are negative (hence digital). With use of Poisson statistics, absolute target concentration can be calculated by the taking into account of all individual reactions tested. When the idea emerged in 1992 [36], these reactions were individual PCR assays in tubes or wells on a plate. The same principle was applied, when the term “digital PCR” was first mentioned in 1999 by Vogelstein and Kinzler [37], who added fluorescent reporters to a PCR. Today, with dPCR, the principle is the same, but the sample does not need to be at limiting dilution. The sample is divided into several small partitions with the help of microfluidics (hundreds, thousands or with some platforms even millions of partitions). Each partition acts as an individual reaction and is exposed to a standard PCR and finally scored as positive or a negative. The ratio between positive partitions and all counted partitions is used in the calculation of the initial target concentration, with use of the Poisson distribution [38]. This technology is being widely adopted for absolute quantification in different areas of research and diagnostics [39–48], including in the field of GMOs, which is further described and discussed in the following text.

## Digital PCR offers several advantages over other PCR-based methods

In comparison with conventional end-point PCR and qPCR, dPCR has a number of advantages. The biggest advantage is the capacity of dPCR for absolute quantification of a target without reference to a standard/calibration curve. This minimizes the effect of matrix differences between the calibrant and the test sample, which could cause different amplification efficiencies [49–51]. Because of the principle of high-level sample partitioning, the results obtained with dPCR are very precise [50, 51] and accurate even at very low target copy numbers [52]. Sample partitioning also allows reliable detection of rare targets in a high background of non-target DNA, which is important for GMO analysis, where a transgene (GMO event) might be present at much lower concentrations than the reference gene (endogene). Another important advantage of dPCR is its lower sensitivity to PCR inhibitors. Finally, an important aspect of routine analyses is cost-efficiency. Although analysis of GMO samples by simplex dPCR is more expensive, the use of multiplex approaches moves the scales in favour of dPCR [53, 54].

## Digital PCR systems used for GMO detection

Several dPCR platforms are available (Table 1) and generally they can be divided in two groups: droplet dPCR (ddPCR; emulsion based) and chip-based dPCR (cdPCR; microfluidic) [55]. For two ddPCR platforms (Bio-Rad’s QX100/QX200 and RainDance’s RainDrop) the reaction mixture is divided into several individual droplets (thousands to millions). Each droplet is amplified by PCR cycling, and amplified droplets are transferred to the droplet reading instrument to determine the number of positive and negative droplets. The RainDrop system provides higher sensitivity (can detect very low concentrations), with millions of droplets generated per sample. The QX100/QX200 and RainDrop platforms are widely used for absolute quantification of GMOs [49, 53, 54, 56, 57, 69, 77]. The QX100 and QX200 systems create around 20,000 droplets per well and have a relatively high throughput (96-well plates are used) compared with the RainDrop platform, which allows analysis of only eight reactions at a time, which are in turn divided into millions of droplets.

**Table 1:** Examples of digital polymerase chain reaction platforms available

	BioMark HD/EP1 (Fluidigm)	QuantStudio 3D (Life Technologies)	Constellation/Constellation modules (Formulatrix)	Clarity (JN Medsys)	Naica (Stilla Technologies)	RainDrop (RainDance Technologies)	QX200 (Bio-Rad)
Partitions	765 or 770	20,000	8000 or 32,000	10,000	30,000	5 × 10^6^–10 × 10^6^	20,000
Total reaction volume (μL)	4–8	14.5	10	15	20	25–50	20
Samples per run	12 or 48	24	96 or 24	32	12	8	96
Duration (h)	~4	~3	~1.5	~4	~2	~7–8	~6
Dyes	FAM/EvaGreen, VIC, ROX (Cy5)	FAM/SYBR, VIC, ROX	5 (FAM/EvaGreen, HEX, ROX, NED, TED, Cy5) for Constellation or 8 different wavelenghts for Constellation Module	FAM/SYBR/EvaGreen, VIC/HEX	FAM, Cy3/VIC/HEX, Cy5	FAM, VIC	FAM/EvaGreen, VIC/HEX
Master mix	Open	Proprietary	Open	Open	Proprietary	Open	Proprietary

In a cdPCR, the reaction is divided into hundreds or thousands of chambers on a single plate or array. The first one that was available, Fluidigm’s Biomark HD, has already been shown to be suitable for GMO analysis [50]. Reports of the use of other chip-based platforms in the GMO field are also available (e.g. Quantstudio 12K Flex [58] and Quantstudio 3D [59], both from Thermo Fisher Scientific). Constellation (Formulatrix) is a plate-based microfluidic dPCR system that offers five-colour multiplexing [60] (Table 1). Clarity (JN Medsys), a relatively new platform, is a chip-in-a-tube technology for sample partitioning, and its performance is comparable to that of the QX100 ddPCR system [61]. The largest difference among cdPCR platforms is in the number of partitions created per sample and in the number of samples analysed in one run (Table 1).

A combination of droplet- and chip-based technology platforms is provided by Stilla’s Naica system for crystal dPCR. It uses a microfluidic sapphire chip, with integration of droplet formation, amplification and readout in a single consumable. It also has three-colour detection, enhancing the options for multiplex dPCR [62]. The Naica system generates more droplets (up to 35,000) compared with the QX100/QX200 system (up to 20,000). However, the input reaction volume is also slightly greater (25 μL compared with 20 μL in the QX100/QX200 system). Analysis is relatively fast with the Naica system owing to a short detection step. However, the Naica system allows analysis of only 12 reactions in one run. There are currently no published reports of the use of the Naica, Constellation or Clarity dPCR systems for the purpose of GMO analysis. This is mostly because they are relatively new on the market, and it is expected that reports on their use will emerge soon. At the National Institute of Biology (NIB), the Naica system has been tested for GMO quantification in both simplex and multiplex format. The performance was comparable to that of the Bio-Rad QX100/QX200 system, with a slightly higher coefficient of variation at low concentrations and lower throughput.

Overall, the currently available dPCR systems are relatively diverse, but nevertheless they are all theoretically suitable for absolute quantification of GMOs. An attempt has already been made to compare different platforms side-by-side [58], and the report showed that all of the platforms produced comparable results. Still, it will be helpful to make further comparisons of different dPCR platforms available for absolute quantification of GMOs, especially on real-life samples and not only on a few selected reference materials. It is important to note here that absolute quantification of individual targets without standard curves by dPCR is in the end translated into a relative value (ratio of transgene versus endogene). Thus, the term “absolute quantification of GMOs” as used in this article refers to the final (relative) percentage of genetically modified content, but from the point of view that absolute quantification of individual targets was used.

## Effect of DNA quality and presence of inhibitors on dPCR

DNA quality is a key factor for successful PCR. The type of samples used, the DNA extraction methods, etc. can affect the quality of extracted DNA (e.g. presence/absence of inhibitors), which can have an impact on amplification with PCR [63]. Demeke et al. [64] reported comparison of seven DNA extraction kits with a cetyltrimethylammonium bromide (CTAB) method for three different genetically modified ingredients: canola, flax and soybean. The extracted DNA was tested with qPCR and the RainDrop ddPCR system. The RainDrop ddPCR system gave more variable results than qPCR. Most of the kits were appropriate for both ddPCR and qPCR for canola and soybean samples, but only one of the seven DNA extraction kits produced consistent results with RainDrop ddPCR for flax samples (Table 2). Canola, flax and soybean DNAs extracted with the CTAB method and purified with a DNA Clean & Concentrator-25 kit were suitable for both RainDrop ddPCR and qPCR assays [64].

**Table 2:** Suitability of DNA extraction kits for quantitative polymerase chain reaction (qPCR) and RainDrop droplet digital polymerase chain reaction (dPCR) for different GM seed samples

DNA extraction method	Canola	Flax	Soybean
Fast ID DNA extraction kit	✓	✓	✓
FastDNA Spin kit	✓	qPCR only	✓
GM Quicker 2 kit	✓	NA	✓
OmniPrep for plant kit	NA	qPCR only	qPCR only
NucleoSpin Food kit	✓	qPCR only	✓
Plant DNAzol reagent	NA	NA	NA
DNeasy *mericon* Food kit	✓	ND	✓
CTAB	✓	✓	✓

Compiled from Demeke et al. [64]. Cetyltrimethylammonium bromide (CTAB)-extracted DNA was purified with a DNA Clean & Concentrator kit

*NA* data not available because DNA extraction was not successful, *ND* not determined (the DNA yield was low and not sufficient for polymerase chain reaction), *tick* worked for both dPCR and qPCR. CTAB extracted DNA was purified with DNA Clean & Concentrator kit

As mentioned already, dPCR assays have been reported to be less sensitive to inhibitors compared with qPCR [57, 65–67]. For samples or target combinations with low levels of nucleic acids and/or variable amounts of chemical and protein contaminants, ddPCR produced more precise and reproducible results compared with qPCR [68]. The reason for this phenomenon lies in the end-point fluorescence reading of partitions. A partially inhibited reaction in an individual partition can still produce a positive signal, and thus there is no or only a little effect on the final quantification result. On the other hand, some inhibitors can still affect absolute quantification by dPCR. One such example is ethanol, which affects both ddPCR and qPCR [57]. For ddPCR, inhibition may be related to chemicals affecting droplet stability (e.g. ethanol) [57], whereas for inhibitors such as EDTA and sodium dodecyl sulfate, inhibition can be asymmetric, with differing extents of assay inhibition in different fluorescent channels [57]. Overestimation or underestimation of a GMO event can occur, if the reference and transgene dPCR assays are not affected by inhibitors in the same way. Thus, this phenomenon can cause issues with GMO quantification, especially if testing is performed with two fluorescent reporters, one for the transgene and another for the endogene. Nevertheless, as reported, this effect is much less pronounced in ddPCR than in qPCR [57]. Overall, it is important to pay attention to the quality and purity of DNA for successful dPCR assays.

**Fig. 1:**
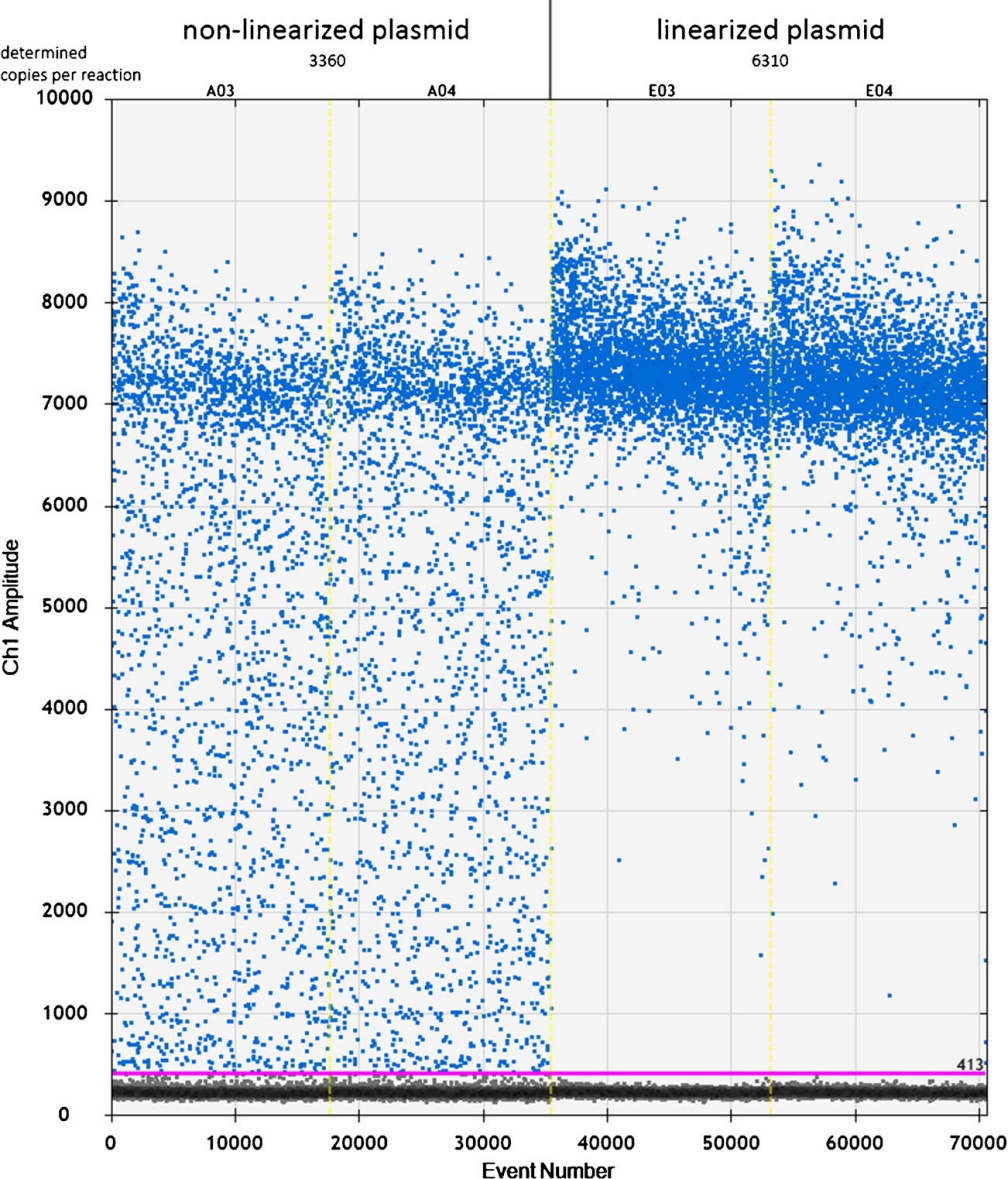
Example of droplet readout from Bio-Rad’s droplet digital polymerase chain reaction system when non-linearized and linearized plasmid are used as the DNA template

Usually manufacturers of dPCR equipment recommend restriction digestion or fragmentation of DNA samples before dPCR assay. This allows separation of possible tandem gene copies and can reduce the sample viscosity and improve template accessibility. Enzymatic digestion of DNA should be carefully planned to avoid any damage in the amplicon region. It is recommended to perform analysis on digested and non-digested DNA samples at the beginning to see the effect on the final quantification. Such an approach was reported for MON810 maize DNA, and it was shown that for the purpose of GMO quantification enzyme digestion was not necessary [49]. Other fragmentation procedures are available besides enzyme digestion. Genomic DNA can be sheared with a Hydroshear Plus® DNA shearing device, a QIAshredder or similar instruments before dPCR [69, 70]. The effect of non-shearing, QIAshredding and hydroshearing of genomic DNA was investigated with a RainDrop dPCR system [71]. The measured GMO percentage values were close to the expected values for three traits at three concentrations in all treatments. Thus, shearing of genomic DNA was not found to be essential for absolute quantification of the GMOs. A dPCR-based method for detection of GMO screening elements, p35S and tNOS, was also reported as appropriate without pretreatment of DNA [72]. Overall, fragmentation of genomic DNA using enzymes or other means may not be necessary for absolute quantification of GMOs as reported for the QX100/QX200 system or the RainDrop system. On the other hand, restriction digestion to linearize plasmid DNA is an absolute necessity [73, 74], as the assay performance and final quantification may be greatly affected (up to two times difference in a determined concentration) because of unavailability of the target in the closed plasmid structure. Problems with unrestricted plasmids can easily be detected on the droplet readout, as unrestricted plasmid can produce a lot of partitions with intermediate fluorescence, and there are no clear clusters of positive and negative partitions (Fig. 1).

## Optimization of dPCR components

The amount of DNA used for dPCR can differ according to the instrument used and the sensitivity required. For example, up to 1000 ng DNA can be used for RainDrop ddPCR to detect a low concentration of genetically modified materials [69], and 100 ng DNA has been used for the QX100/QX200 system and other systems. Generally, the same amount of DNA used for real-time qPCR can also be used for dPCR. At NIB, DNA quantity is usually assessed by means of a preliminary qPCR run targeting plant endogenes. Our experience has shown that spectroscopic measurement is not accurate enough. From comparison of measurements with NanoDrop, Qubit and ddPCR, it was observed that NanoDrop overestimated the quantity of genomic DNA by more than two times and Qubit overestimated it by around 50% when compared with the ddPCR results. For cdPCR (e.g. Fluidigm), the assessment of DNA quantity in a reaction is more problematic than in ddPCR because of the narrow dynamic range. Independent of the quantification method, it is important to ensure there are non-denaturing conditions for DNA samples because the quantification result for double-stranded DNA can differ from that for single-stranded DNA by 100%.

The manufacturer-recommended concentrations of 250 and 900 nM for the probes and primers, respectively, usually perform well for ddPCR assays. However, optimization can in some cases reduce the concentrations to limit the amount of primers and probes used. Nevertheless, more thorough optimization of primer and probe concentrations is especially important for multiplex dPCR assays. Probes are generally labelled with FAM, HEX or VIC, and non-fluorescent black hole quenchers are generally used. If multiplexing is performed in one fluorescent channel, the concentration of probes and/or primers should be optimized thoroughly to allow clear separation of clusters [56]. It is also important to combine primers that do not interfere among themselves for multiplex assays. Testing primer/probe interactions may not be sufficient, and thus actual wet laboratory experiments must be performed to assess the performance of multiplex assays [53, 54]. Careful experimental design is of great importance to identify such interactions, and an initial large amount of work can save time at later stages.

For PCR, an annealing temperature of 60 °C is generally used. The annealing temperature has an effect on the resolution between clusters of positive and negative partitions (resolution is increased by lowering of the temperature). However, one must be extremely careful with lowering the annealing temperature as non-specific products can be amplified [75]. Luckily, positive partitions as a result of such non-specific amplification can be distinguished from real positive partitions on the basis of their fluorescence amplitude. For thermocycling, the ramp rate can also be important. At first, Bio-Rad’s general recommendation was 2.5 °C/s; however, the latest recommendation is to use 2 °C/s, which increases cluster resolution and reduces the number of droplets with intermediate fluorescence. To increase the resolution between clusters of positive and negative partitions, the total number of PCR cycles can be increased, but in such a case again non-specific amplification can occur.

The performance of an assay for the purpose of GMO detection and quantification must, at least in the EU, be characterized and needs to comply with the minimum performance parameters [27]. Parameters such as accuracy, repeatability, robustness, limit of detection and limit of quantification are thus usually reported with each newly developed assay. According to Vynck et al. [76], simple calculations of linearity and high *R*^2^ values may not necessarily show suitability for PCR; thus, they suggested a robust weighted least squares approach as a suitable alternative [76].

One of the most crucial steps in Bio-Rad’s ddPCR workflow is the transfer of the fragile freshly generated droplets from cartridges to the PCR plate. According to the manufacturer’s recommendations, the transfer of generated droplets with a constant low pipetting speed (suction) and an appropriately steep angle of filter tips gently touching the microtitre plate wall helps to minimize mechanical disruption of the droplets. The amount of accepted droplets can increase with practice and pipetting optimization [77], but perhaps the best solution for a controlled and repeatable pipetting procedure is, at least for Bio-Rad’s QX100/QX200 ddPCR system, the use of a automated droplet generator. The pipetting robot handles all of the pipetting steps, from pipetting of the reaction mixture into cartridges to pipetting of the droplets onto the PCR plate.

## Digital PCR for absolute quantification of copy number and the factors affecting the results

In principle, as already mentioned, the absolute target concentration in a sample is calculated on the basis of number of positive partitions and all accepted partitions and by use of a Poisson distribution [38]. The final absolute target concentration in a sample is calculated according to Eq. 1:

1 $$ {T}_{\mathrm{c}}=-\ln \left(1-\frac{P}{R}\right)\times \left(\frac{1}{V_{\mathrm{d}}}\right)\times D, $$

where *T*_c_ is the mean target concentration (copies per microlitre), *P* is the number of positive partitions, and *R* is the number of partitions analysed. As can be seen from Eq. 1, the target concentration also depends on the partition volume (*V*_d_). A dilution factor for the original sample before PCR (*D*) is also considered in the equation. Essentially, three factors affect the final result: (1) correct classification of partitions as positive, (2) correctly determined partition volume and (3) a dilution factor.

### Classification of positive and negative partitions

Fluorescence readout is performed for most dPCR platforms with a dedicated machine after PCR or the fluorescence of partitions is measured in each amplification cycle. Finally, the raw result is the fluorescence amplitude of each individual partition. Digital PCR (dPCR) providers offer software for visualization of fluorescence readout; however, raw results can also be analysed independently with use of other tools (e.g. R [78]). Classification of droplets as positive or negative is usually not an issue when it is performed visually by the software provided. An automatic approach was shown not to be the best option, especially in the case of assays where partitions with intermediate fluorescence are abundant. These partitions can contain target DNA, but the reaction may be less efficient [43], or they can be false positives; thus, it is important that they are classified correctly. On the other hand, manual threshold setting can be affected by the operator’s subjective decision. Thus, specifically designed automated approaches, which take into account the distribution of positive and negative droplets and implement statistical significance [75, 79], might be the best option to increase repeatability and reduce bias between operators.

To facilitate more reliable classification of partitions, the assay must be optimized in a way that there is as low number of partitions with intermediate fluorescence as possible and that the resolution between the positive and the negative cluster is at least 2 [75]. Nevertheless, our experience has shown that less than optimal assays can still produce reliable absolute quantification results. The reason for this lies again in the fact that partitions with intermediate fluorescence can also be scored as positive.

### Partition volume

The partition volume is one of the most critical factors affecting target absolute concentration calculation. Discrepancies between partition volumes assigned by the manufacturer and measured in independent laboratories have been reported for ddPCR platforms [38, 58, 80–82]. At NIB, Bio-Rad’s QX100/QX200 platform has been tested most rigorously. The first version of QuantSoft used by the Bio-Rad QX100/QX200 platform considered a volume of 0.91 nL in the calculation, but the volume was later measured to be 0.868 nL [38] or 0.834 nL [80]. These measurements were performed with a now discontinued line of cartridges. With a new line of cartridges, the software was updated to consider a volume of 0.85 nL. Nevertheless, measurement of the new cartridges showed a deviation from Bio-Rad’s default value, with volumes of 0.767 nL [81] and 0.715 nL [82]. The most recent study [82] not only showed that the volume of droplets is significantly lower but also that the volume of droplets is affected by type of super mix (for probes or EvaGreen) and the type of droplet generator (manual or automated). More interestingly, when Bio-Rad’s default droplet volume was used at NIB, lower absolute target concentrations were determined by ddPCR (Bio-Rad) compared with cdPCR (Biomark HD) not only for GMO samples (unpublished data) but also for other samples, such as human cytomegalovirus [83]. The values obtained with the ddPCR and cdPCR platforms were much closer when correction-factor-based measurement was performed [82]. This phenomenon was also observed for the RainDrop platform. The droplet volume was corrected on the basis of the droplet measurements, and the measured volume of 4.39 pL was used to calculate the target concentration instead of 5 pL provided by the manufacturer [58]. As concluded by Bogožalec Košir et al. [82], it is of great importance to know the exact droplet volume, which might even be laboratory specific.

Accurate dPCR quantification is important in clinical and diagnostic decisions, and thus it is important to at least consider the possibility of incorrect volume and implement this in the calculation of expanded measurement uncertainty. However, in the GMO testing field, the final result is a relative genetically modified content, with the ratio of transgene to endogene, and as such an incorrect droplet volume would affect the absolute copy number of the transgene and the endogene in the same fashion, and thus the relative ratio would not be affected.

### Dilution factor

In dPCR, samples are usually diluted before the reaction because of the limited dynamic range. The target concentration in the stock material is then calculated with use of dilution factors, factors by which the sample was diluted before dPCR. To minimize uncertainty due to pipetting and not to compromise the accurate absolute measurement of copies by application of an incorrect dilution factor, the best approach is to perform all pipetting steps, including preparation of DNA dilutions and reaction mixtures, gravimetrically, with use of calibrated mass balance. This approach was used in studies with the purpose of showing accuracy of measurements with dPCR and to minimize the measurement uncertainty [38, 50, 58, 80, 84]. The drawback of this approach is the extent of additional labour and calculations based on the measured masses. Thus, this approach is usually used only for studies where accuracy is of utmost importance (e.g. stability studies of reference materials or even certification of reference materials). Nevertheless, it is beneficial, if a laboratory assesses the procedure by occasionally implementing a gravimetric approach to control the possible error. At NIB, the effect of pipetting error was assessed by comparison of the gravimetric and volumetric approaches. The difference between the results was around 0.5%. Of course, the error is operator and pipette dependent, but once this uncertainty is assessed and controlled, there is no need to use a gravimetric approach for further analyses.

## Multiplex quantification of GMO events with dPCR

Multiplexing is readily available in dPCR systems, as all platforms include filters that allow detection of fluorescence in at least two channels (FAM and HEX/VIC). Some have the option of even higher multiplexing, because of the availability of filters for up to five fluorescence channels (Table 1). Duplex absolute quantification is very suitable for GMO analysis, as transgenes and endogenes can be quantified in the same reaction, and thus it can be easily implemented into the testing scheme. Morisset et al. [49] reported on the suitability of duplex reaction for quantification of MON810 transgenic maize. To test the transferability of such a protocol to other laboratories, one DNA sample was tested in three independent laboratories in the Decathlon project (http://www.decathlon-project.eu). The results showed good comparability of determined values between laboratories in terms of absolute copy numbers determined for each target and GMO content (Table 3).

**Table 3:** Absolute copy numbers for stock DNA for MON810 and hmgA target determined by three independent laboratories on the same DNA sample by duplex droplet digital polymerase chain reaction and calculated genetically modified (GM) content

Target	Laboratory 1	Laboratory 2	Laboratory 3	Coefficient of variation (%)
MON810	2768	2775	2951	9
	2282	2409	2599	
hmgA	76,874	71,213	80,616	11
	58,566	62,665	76,312	
GM content (%)	3.73 ± 0.21	3.87 ± 0.25	3.54 ± 0.22	4.5

The results for two dilutions, each tested in duplicate, are presented for each target. The results for GM content are presented as an average from all replicates together with the 95% confidence interval

The choice of the reporters for TaqMan probes is limited by the availability of only a few fluorescence detection channels (usually FAM and HEX/VIC). Therefore, a higher level of multiplexing in dPCR can be achieved by other approaches, summarized by Whale et al. [85]: amplitude-based multiplexing, ratio-based multiplexing, ratio-based non-discriminating multiplexing, and non-discriminating multiplexing. These approaches involve modifications of probe concentrations for individual targets or use different ratios of fluorescent reporters for individual targets. These modifications allow spatial separation of respective clusters of amplified targets on the basis of their fluorescence level. Two of the approaches (non-discriminating multiplexing and amplitude-based multiplexing) were reported for GMO quantification [53, 54, 56]. In the EU, Regulation (EC) No 1829/2003 specifies that quantification of the concentration of genetically modified material should be per ingredient (interpreted also as per species). Consequently, a non-discriminating multiplex approach with two reporters can be used to quantify the species-specific reference gene in one channel and all of the authorized GMOs belonging to that same species simultaneously in the other channel. Such an approach was reported for maize [54] and soybean [53]. Although this is not common practice in the EU enforcement laboratories, such an approach can be used for testing legal compliance of EU-authorized GMOs. Another approach, amplitude-based multiplexing, allows quantification of four individual targets simultaneously in one reaction, but has some limitations in terms of specificity when highly concentrated DNA samples are used, because of the possible presence of droplets with intermediate fluorescence [56].

## Summary and future prospects

Digital PCR (dPCR) is being used for a wide range of applications in medical, environmental and agricultural areas. The most obvious advantage of dPCR is the possibility to obtain accurate absolute target concentration with no standard curve requirements. Selection of the proper dPCR instrument for a particular laboratory’s need is important. Currently available dPCR instruments seem to be suitable for quantification of GMO events. However, there is variation in throughput and sensitivity, and thus laboratories should assess their needs and budget before making a final decision. Assays used for qPCR can readily be transferred to dPCR; nevertheless, some optimization of the primer and probe concentrations might potentially improve the overall assay performance. A more thorough evaluation and/or verification is essential, especially for assays used in a multiplex dPCR format. Many reports have shown that dPCR is less sensitive to inhibitors compared with qPCR, indicating that it might be a method of choice for samples where the presence of inhibitors is expected. It has also been reported that the DNA extraction method used and DNA quality affect dPCR results. Care must also be taken with partition or droplet volumes assigned by the manufacturer, as the actual values could differ and adjustments may be necessary. As the final GMO content is presented as a relative value, some factors which affect absolute quantification can be partially ignored if they affect both the target and the endogene in the same way. The advantage of using absolute quantification of GMOs (transgene and endogene) is in the elimination of the need for a standard curve generated from certified reference materials. Cost-efficiency is currently still on the qPCR side for simplex assays; however, multiplex assays shift the cost-efficiency towards dPCR.

Digital PCR (dPCR) has the potential to replace real-time qPCR. Further research on evaluation of different dPCR instruments and collaborative studies to confirm the wide applicability of the system will thus be useful. Another challenge is the inability to detect unauthorized GMOs with current DNA-based technologies. Genome-edited plants are also gaining popularity. Genome editing allows the introduction of insertions, deletions and substitutions at predetermined sites in the plant genome with use of designer nucleases (e.g. CRISPR/Cas9) [86]. Although, no decision has been made on the regulation of products obtained by new breeding technologies so far, the European Commission prepared an explanatory note on these techniques [87]. It seems that identification and quantification of genome-edited plants will be a challenge if they become regulated in some countries. PCR technologies might be replaced by NGS in the future, especially for identification of genome-edited plants. However, identification of unknown single SNPs (as a result of genome editing) might still prove difficult because of the natural occurrence of SNPs in the genomes. Thus, it will be helpful to develop a strategy for the detection of genome-edited plants in case they become regulated in some countries. Nevertheless, the quantitative aspect is still far away for NGS, and therefore we can expect that dPCR will be a leading technology for this purpose for some years to come. At NIB, ddPCR has been implemented into routine testing for official control, with five ddPCR assays within ISO17025 accreditation. On the basis of NIB’s example, it is expected that more laboratories will follow with implementation of ddPCR for routine GMO analyses.
